# How to perform an excellent radiology board examination: a web-based checklist

**DOI:** 10.1186/s13244-020-00924-0

**Published:** 2021-01-07

**Authors:** Oğuz Dicle, Sema Özan, Hatice Şahin, Mustafa Seçil

**Affiliations:** 1 Department of Radiology, School of Medicine, Dokuz Eylül University, İzmir, Turkey; 2 Department of Medical Education, School of Medicine, Dokuz Eylül University, İzmir, Turkey; 3 Department of Medical Education, School of Medicine, Ege University, İzmir, Turkey

**Keywords:** Board examinations, Radiology education, Assessment standards

## Abstract

**Background:**

Board exams are now considered as means of quality procedures that aim to keep the professional knowledge and skills of the physicians at the highest level. In addition, for an assessment to be scientifically valid, it has to be done within defined standards. Although there are different sources in this field, there is a need for a resource that details the steps required for the examinations to be performed perfectly, brings descriptions of the reasons for the procedure and associates the steps with assessment standards. Experts with national and international experience both in radiology and medical education contributed to the preparation of this checklist.

**Results:**

The guide includes 174 elements to consider before, after the exam order and examination. From the perspective of assessment standards, it has been observed that the steps to be considered before the exam have a greater impact on the validity and reliability of the exam. The standard in which the questions are most associated was validity with 117 (67.24%) questions.

**Conclusions:**

We think that our guide, which will be accessible in the web environment, will be useful to the teams with a development goal or just start the exam, the candidates who will take the exam and the examiners.

## Key points

- There are many steps to pay attention before, during and after the exam.
- A checklist with explanations helps to create a common language.
- Assessment standards should always have to be checked for each step.
- Availability from the web is an advantage for the use of the guide.

## Background

In medical practice, it is essential to provide a safe and high-quality service for patients, which is only possible with the help of quality assurance that covers a spectrum of standards in medical education, healthcare centers and medical devices. Total quality management, standardization, certification and accreditation are some of the tools of these processes. Additionally, utilizing guidelines in diagnosis and treatment management helps to enhance the quality of medical care. However, the most important of these is to ensure that the knowledge and skills of the physicians are constantly updated. It has become more than an issue that the knowledge and skills after the physician’s graduation decays and health professionals have limited ability to assess themselves [1, 2]. On the other side, patients usually assume that their physicians follow the recent medical improvements [3]. Many different attempts have been made to overcome this situation. Proficiency exams, logbook applications, obtaining continuing medical education credits, in-service training applications, maintenance of certification programs (MOC) used in the United States of America (USA), and board exams are some examples of these initiatives. Board exams have met the requirement in this area to a large extent and have been a good tool for evaluating the knowledge skills and competencies of physicians, especially in their fields of expertise.

Board examinations in various medical specialties are being applied, some of them have a history of a century. Early organizations started in the USA. This is followed by the unification of specialty boards as the Advisory Board for Medical Specialties in 1933. It was later renamed as the American Board of Medical Specialties (ABMS). Traditionally, the model used in the USA has been self-regulation to hold the profession accountable to the public and is seen as a privilege. The content, method and evaluation concepts of the board examinations have been dynamically changed over the years, and a huge experience has accumulated for the rest of world [4].

The American Board of Radiology (ABR) was established in 1934 after negotiations between the representatives of the American Roentgen Ray Association (ARRS), the Radiological Society of North America (RSNA), the American Radium Association (ARS), and the American College of Radiology (ACR). Later, the Radiology Department of American Medical Association (AMA) also joined as a sponsor. Over time, the American Association of Physicists in Medicine (AAPM), the American Society for Radiation Oncology (ASTRO), the Association of University Radiologists (AUR), and the Society for Interventional Radiology (SIR) have also been added to ABR. The first examination and certification of the board for medical physicists was carried out in 1947. Along with the developments in radiology, there were also certifications added or deleted. In 1994, some sub-specialization areas were introduced and after 2002, “time-limited certificates” were completely replaced. In 2015, progressive removal of “written” and oral diagnostic radiology exams and transition to computer-based diagnostic radiology was completed for initial certification exams. Online Longitudinal Assessment (OLA) was introduced in 2019, which could replace the Maintenance of Certification MOC Exam [5].

The most important actor in the history of board exams in Europe is the Council for European Medical Specialty Assessments (UEMS-CESMA). UEMS-CESMA was established in 2007 within the body of the European Union of Medical Specialists (UEMS), consisting of 50 affiliated UEMS boards and societies. Its aim is the recognition of European Postgraduate Medical Assessments (EPMA) as the European gold standard. In order to achieve this, harmonization of evaluation standards is one of its main objectives. The first European Diploma Exam is the European Diploma of Anesthesiology (EDA) held in 1984. Today, more than 30 disciplines of European board exams are being held, including Radiology [6, 7].

The European Board of Radiology (EBR) was founded in 2011 by the European Society of Radiology (ESR). EBR, which has been officially approved by the European Union of Medical Specialists (UEMS), has aimed to develop standards that will reference the diverse Radiology specialty training programs and exams in European countries. It works to ensure the standardization and accreditation of radiologists in Europe and other demanding countries. Certified radiologists and resident who are in the last year of radiology education programs in their own countries are accepted to European Diploma in Radiology (EDiR) exams, and the examinations are being held worldwide throughout the year [8, 9].

In general, testing one’s competency within 10 years period has been accepted for practical reasons [10]. These evaluations mainly concern to assess the professional knowledge and skills that physicians have to gain. Written, oral and skill examinations are used as well as credit collection and log-book systems on certain practices. In recent years, computer-based assessment methods have become more popular and common [11].

Concerning the postgraduate education and the assessment of competency in medical specialties UEMS, World Federation for Medical Education (WFME), Accreditation Council for Graduate Medical Education (ACGME), American Board of Internal Medicine (ABIM) and General Medical Council (GMC) guides are accepted to be the most dedicated documents that is widely used for this purpose. There are many standards set for examination and evaluation systems in these documents. Among these, six criteria can be said to come to the fore. Validity and reliability is the most known of these standards. Validity and reliability is a *sine qua non* and the key quality parameter [10, 12–21]. Validity and reliability of the high-stakes examinations are particularly important because of the legal justifiability. Practicality, cost, fairness and educational impact are the other terms and parameters that are used to evaluate the value and the quality of the assessment [10, 12–16, 20–22]. Although there are other definitions such as feasibility, acceptability, utility which have similar meanings with these parameters, it could be thought that the criteria defined above will meet the general [10, 12–16, 20, 21, 23]. However, in most of the cases, the content and description of the parameters interfere with each other. In order to speak about an excellent assessment, it has to be handled and checked in terms of assessment standards.

The standards, guidelines, and recommendations that are recommended to be followed for assessment in many sources are already available. Most of these documents frame a general standard for the application of the examination. However, there is no such a specific guideline that provides a detailed key involving all the steps of a competency-based board examination, also supplying the explanation of each item with the related literature.

Our study mainly aims to fill this gap and provide a scientific and instructional tool to those who perform, implement, and practice these examinations. For this purpose, it is aimed to define all the required steps to conduct an excellent board exam and to show the relationship of each step with the assessment standards. With the help of our checklist which will be accessible on the web, the candidates will benefit to improve their performances and enhance their consciousness about their evaluation. In this study, radiology, as a domain, is a sample for the general process. Newly established boards, independent from the domain, and those who aim to improve and standardize their board examinations will benefit from this tool.

## Materials and methods

### Preparation of the item pool

Aiming to determine the widest possible item steps to support the content validity of checklist, we selected the articles, textbooks, guidelines of the authors, related associations, etc., in the field of medical education and evaluation among many sources containing similar and/or overlapping information. A large Google research was carried out using the key words of “board examination, assessment, assessment standards, assessment checklist, assessment form, certification, validity, reliability, fairness, educational impact, cost, practicability and utility”. In addition, the websites of medical associations, EBR and different medical discipline associations [UEMS-CESMA, WFMA, ACGME, GMC, Association for Medical Education in Europe (AMEE), ABIM, American Educational Research Association (AERA) etc.] have been searched and related documents, guidelines, reports were listed. Textbooks written for medical education and assessment were also utilized to harmonize the terminology. The sources we used were between 1997 and 2020, and the language was English.

A comprehensive 190-item expression pool consisting of stages, steps, and actions that are considered important in carrying out an Excellent Board Examination was created.

### Writing explanations for each item

During the literature review, while determining the expressions to be included in the pool, the importance of them in terms of evaluation/board, and explanations regarding why they would be necessary were also examined.

Besides the resources, the expertise and experience of the researchers were used in the preparation of the explanations regarding the questions. Then, in order to make it clear to the users of the checklist, an additional column of explanation was added for each statement. It is thought that the explanations will contribute to create a common language, to increase comprehensiveness, and to practice with higher awareness.

### Transforming expressions to questions

After the draft checklist was developed, expressions were converted into question phrases that could be answered as "yes"/"no". The purpose of this was to understand whether the quality standards related to the statement/question have been met, since it could be determined whether the specified activity/action/step has been carried out when the checklist has been put into use. For this purpose, a related column is included in the checklist after the question and explanation columns.

### Grouping questions and editing under related topics

The questions in the item pool are grouped under four main titles, considering all the steps related to a board exam: (1) management of the entire procedure: general principles, (2) before examination/preparation for the exam, (3) implementing the exam and (4) after the exam. It has been observed that the questions under each title could also be collected under subtitles and the relevant subtitle names have been determined. The titles and subtitles are indicated in Table 1.

**Table 1:** Checklist titles and subtitles

1. Management of the entire procedure: general principles	
2. Before exam/preparation of the exam	
a. Establishment of examination committee	
b. Identification of eligibility criteria for the candidates	
c. Application procedures	
d. Exam date, location, staff and materials	
e. Blueprint	
f. Question writing and collecting process	
g. OSCE*-type stations (preparation, set up, etc.) (optional)	
h. Oral examination (optional)	
i. Preparation of Simulated/Standardized Patients (SP)/Cases (optional)	
j. Audit of questions	
k. Question booklets/Display	
l. Scoring	
m. Setting the pass mark	
n. Informing about the exam	
o. Preparation support for the candidates	
3. Implementing the exam	
a. Before starting	
b. During the examination	
c. At the end of the examination	
4. After the exam	
a. Wrap up	
b. Right of objection	
c. Reading and marking procedure	
d. Test/item analyses	
e. Declaration of the results	
f. Appeal process	
g. Evaluation of the results	
h. Quality development	

*OSCE Objective structured clinical examination

### Determination of the assessment standards

The standards that will be taken as a basis for performing a perfect evaluation are determined by the literature review. There are many standards in the literature that are expected to be considered in an assessment [10–25] which can be handled individually or together. In our study, six basic standards agreed upon by the researchers were determined as the recommended standards to be controlled during the assessment process. These standards were practicability, validity, reliability, cost, fairness, and educational impact. A description of the standards is given in Table 2. The columns related to the standards are listed in the end of the check list.

**Table 2:** Assessment standards for assessment procedures

Practicality	Practicality refers to the extent to which an assessment or assessment procedure is easy to administer and score. It concerns the adequacy of resources and how these are allocated in the design, development, and use of assessments. Resources to be considered are human resources, material resources, and time [24, 25]
Validity	Validity is defined as the extent to which an assessment accurately measures what it is intended to measure [12, 13, 18]. Almost everything in assessment is related with validity in some way [18]
Reliability	Reliability is the degree to which an assessment tool produces stable and consistent results [10, 13, 14, 19]. Validity and reliability are closely related. Although the reverse is not valid, reliability is a prerequisite for the validity of an evaluation [13, 26]. For this reason, all items associated with reliability in the checklist are also matched with validity
Cost	Cost effectiveness/cost, which is an important feature of evaluation in terms of feasibility and utility, is one of the features suggested in evaluation methods [10, 15, 20]
Fairness	Fairness is to be careful and fulfill the requirements of equality, diversity, disability, gender, sensitivity in terms of cultural aspects, preventing bias, openness in the expectations expected from candidates in all processes of evaluation [15, 18, 22, 23]
Educational impact	It is a standard that directs the learning behaviors and methods of the candidates and defines the impact of assessment on learning [12, 14, 19]

### Matching questions with assessment standards

Information related to the standards was noted at the literature review step to determine the draft statements. Therefore, matching the questions in the draft checklist with the standards was determined based on the literature review and the researchers' expertise and experience. As a result, each question was matched with the standard, whose relationship was considered the most obvious. Two of the researchers were radiology professors experienced in board examination management more than 20 years. Two of them were professors in medical education with an expertise in assessment and evaluation. Each question is matched to the standard to which it relates directly. "√" sign is used in the matches. The process was carried out by seeking consensus if the parameters assigned blindly by the two experts showed any conflict. The results were then evaluated independently by the other two authors, and in case of disagreement, a decision was made to be eligible in a collective discussion environment.

As a result of the transactions made up to this step, a draft checklist consisting of 8 columns in Microsoft Excel format with questions, explanations and standards was created (Table 3).

**Table 3:** A sample from the checklist with the items, explanations and matched assessment standards

Items	Explanations	Practicality	Validity	Reliability	Cost	Fairness	Educational impact
*Management of the entire procedure: general principles*							
Is the examination process handled independently from the main body of the related society?	Board examinations should be done in affiliation with the Specialty/Subspeciality Societies. This can be handled by an external professional office or a volunteered group of education committee members. However, the process and the management have to be carried out independently. Society may only give a financial and manpower support. Ideally, the Board Examination should be organized by the (national) entity that is responsible for the issue of the Radiology Certification. Independently from any organization that may have potentially related conflicts of interests. In countries having more than one society, the association that has been recognized by international organizations and/or the association who has higher number of members is recommended to be responsible from the Board examination				√	√	
Is the examination organized without any financial profit?	Board examinations should be non-profit organizations. Fee is required to finance the exam outcomes and the professional services					√	
Is the examination carried out with a team of different people with different qualifications?	An office with dedicated members, IT persons and an examination committee with experts in the field of education, examination and assessment has to be established [27]	√	√	√			√
Do you make sure that the management is done professionally?	The main society has the responsibility to make sure that the examinations are done professionally. Executive committee of the related boards should evaluate the procedure and the team continuously [10]		√	√			√
Do you manage the process through an instruction?	An instruction including the main goals, the details of the Board Exam process, evaluation procedures, role of the people and the outcomes is recommended	√	√	√			
Do you prepare an overall timeframe for each examination?	A timeframe including the schedule of all steps of examination has to be prepared. Dates, responsible persons, deadlines and the places to be used should be taken into consideration [27]	√					
Is all the process of the examination correctly blinded and anonymized to guarantee fairness and equity?	Anonymization of personal data should be respected during all the process (examination, handling of data, correction and publication of results)					√	
Are all personal data correctly handled respecting the individuals' rights and accordingly to the current laws?	Management of personal data should be done accordingly to the current laws respecting the individuals' right					√	
*Before exam/preparation of the exam*							
Establishment of examination committee							
Do the exam committee members hold a board certificate?	It is recommended that the exam committee members should have a board certification. Another option is to choose the committee members among medical specialists who have experience in education and assessment. This option is recommended especially If the board examination will be done for the first time [28]		√	√			
Are they in good standing with the profession?	The most important criteria in this aspect is the ethical interest of the individual [28, 29]		√	√			
Are they currently active in clinical practice?	Examiners who are in active clinical practice assumed to be aware of recent methods and terminology [28, 29]		√	√			√
Did they received an assessment training?	Certified examiners on assessment will increase the quality of the examination which makes it more reliable and valid [29]	√	√	√	√		√
Do they have good written and verbal communication skills?	Good written and verbal communication skills may deeply affect the examination. It is recommended that the exam committee members should have these capabilities. International examinations have some additional difficulties related with communication. However, it is not easy to decide whether the examiner has good written and verbal communications skills. A convenient solution will be the use of feedbacks collected during the quality assurance process [28, 29]	√	√	√			

### Expert reviews

In order to support the content validity of the checklist, expert opinions were additionally consulted. For this purpose, the first two columns with questions and explanations in the draft checklist shown in Table 3 were sent to 10 experts from different countries with board examination experience via e-mail accompanied by a letter. By explaining the purpose of the study, the letter asked the experts whether each title, question and explanation was appropriate for the radiology board exam process, whether they suggested adding or removing them, and if any, they were asked to specify. A second reminder was made at the end of two weeks. At the end of a month, comeback was received from eight experts. Suggestions for the explanation of 54 questions came and related explanations were added. Ten new question suggestions came, questions and related explanations were also added. In the revised checklist after receiving the expert opinion, the title and subtitles were preserved, and the number of questions was clarified as 174. Together with the authors of the article, the opinions of 12 experts were used in the formation of the checklist.

### Data analysis

Distribution of suggestions given by the experts was listed, and the percentage of suggestions according to main titles was calculated. Distribution of finalized questions according to titles was listed. Descriptive statistics (percentage, frequencies) to evaluate the impact of standards were used.

### Web tool development

The checklist we developed has been turned into a web application that will be used by those who want to improve their board exams in line with the standards, and especially those who plan to take a new board exam. It is thought that the explanations about each question will facilitate those who will use this tool. Users will also be able to see how many questions they can answer as yes using this tool. The relevant web tool can be accessed at https://medinfo.deu.edu.tr/checklist/ [12–16, 18, 19, 27–63].

## Results

During the preparation of the checklist, opinions of twelve experts experienced in board exam planning and applications were received. Accordingly, feedback was received on 54 questions, and add-subtract suggestions were made. The distribution of the suggestions according to the main title in the checklist is given in Table 4. It was seen that most of the suggestions (62.96%) were related to the “Before exam/Preparation for the exam” part of the check list.

**Table 4:** Distribution of suggestions by main title

Main title	Number of suggestions	%
Management of the entire procedure	6	11.12
Before exam/preparation for the exam	34	62.96
Implementing the exam	7	12.96
After the exam	7	12.96
Total	54	100.00

The findings related to the checklist prepared for the board examination planning and implementation are presented below. Checklist consisted of four main titles. There were subtitles and question sentences under the main headings (Table 5). The checklist consisted of a total of 174 questions under 26 subtitles. “Before exam/Preparation for the exam” main title had the maximum number of intermediate titles and questions.

**Table 5:** Distribution of subtitles and questions according to the main titles

Main title	Number of subtitles	Number of questions
Management of the entire procedure	–	8
Before exam/preparation of the exam	15	109
Implementing the exam	3	24
After the exam	8	33
Total	26	174

All 174 questions in the checklist were also reviewed in terms of practicality, validity, reliability, cost, fairness and educational impact standards. Distribution of items according to the assessment standards and main titles are given in Table 6. A percentage of 67.24% of the questions met the standards of validity while 60.34% of them met both with reliability and of fairness.

**Table 6:** Distribution of items according to the assessment standards and main titles (N = 174)

Standards	Management of the entire procedure	Before exam/preparation of the exam	Implementing the exam	After the exam	Total n/N (%)
Practicality	3	33	6	5	47 (27.01)
Validity	3	78	18	18	117 (67.24)
Reliability	3	70	18	14	105 (60.34)
Cost	1	16	11	3	31 (17.82)
Fairness	4	56	21	24	105 (60.34)
Educational impact	2	22	1	6	31 (17.82)

Although most of our items match a few standards, only 10 of the 174 items match all five standards concerning practicality, validity, reliability, cost and fairness. When the distribution of these 10 questions was analyzed, half of the questions that meet all the standards were the questions of the “Before exam/Preparation of the exam” subtitle.

## Discussion

Nowadays, keeping the professional standards of medical professionals consistently high has been accepted as a guarantee of good health care and patient safety. Board exams at national and international levels are among the most important applications developed on this acceptance. Board exams, which have been a long past, have been developed and expanded over the years. Medical institutions, national health authorities, specialist associations and organizations, and many institutions such as UEMS and ACCME, whose basic missions are the development of professional standards, have been making various suggestions, guides, training studies and publications for the board exams to be held according to the educational standards. In parallel with this, an important accumulation of scientific literature has also occurred. On the other hand, the experts who have given their years to these exams carry a unique and invaluable experience. Our study aimed at a holistic approach that would enable us to prepare a perfect board exam in the light of this rich experience.

Considering the importance of expert experiences, the opinions of experts with at least 10 years of national and international experience in board exams were used in our study. Long experience of the experts has provided an easy consensus during the review phase and it has been possible to create a complete checklist about the entire exam with their suggestions. Similar results could have been obtained using the Delphi method. However, it was thought that it would be more beneficial for experienced experts to give interactive opinions instead of the mechanical approach based on the questionnaire and scoring of Delphi method, and the data were produced in this way.

As seen in the findings, although all the stages related to the examination process are very important, pre-exam preparations are the stage where the most steps are required. When the steps are evaluated in terms of matching the standards, each standard was included under the heading "Before exam/Preparation of the exam." This finding shows us that the “exam preparation” stage has a special place among all the exam steps and the practices related to this phase must be done meticulously to conduct a perfect exam.

In the developed checklist, the assessment standard that most closely matches the questions at all stages of the exam was validity. Validity is an indispensable feature in terms of defending the comments and decisions reached as a result of the evaluation [10, 18]. It is particularly important to demonstrate the validity of the decisions at the end of the certification exams because the inadequate success of candidates with insufficient competencies will have undesirable consequences for candidates, institutions and healthcare provided [64]. Therefore, the more "yes" answers given to questions that match the validity, the higher the validity of the exam will be.

Following validity in our checklist, the standard that most closely matches the questions was reliability. The goal of evaluation is to achieve the desired level of measurement accuracy and to maintain the consistency of scores over time under different test conditions and different evaluators [10, 12, 13]. High reliability standards of the exam will enable us to achieve these goals. However, it should be noted that reliability is affected by almost all error sources. Effort for reliability also contributes to the fairness and transparency of the assessment [10, 16].

Similar to reliability, fairness has become the standard that most matches the questions. In all processes of evaluation, to ensure the equality and diversity, to be sensitive to the requirements of disability, gender and cultural sensitivity, to prevent bias, to be open about what is expected from candidates, etc. are important points in terms of exam's fairness [15, 18, 22, 23]. Being careful about these issues and fulfilling their requirements will provide candidates with fair opportunities to demonstrate what they know and do, and to be successful [22]. However, in this case, it will be guaranteed that any candidate will not be allowed to gain an unfair advantage over others [16]. Candidates' feeling that the evaluation is fair will also have a positive effect on their motivation [14].

Students determine their priorities in learning according to what they believe will take place in the exams, focus on them and guide their working strategies according to the types of questions they will encounter [12, 14, 15, 26]. It can be discussed whether the educational impact, which is an important standard for all evaluations, is taken into account in board exams. Candidates who will take the board exam are those who have passed undergraduate and (mostly) graduate exams and completed their education. On the other hand, we can predict that the candidates will have a preparatory process again before an exam that will confirm that they are doing their profession at an excellent level, and that these exams are also included in the scope of Continued medical education/Continuing professional development (CME/CPD). In Primary and Final Fellowship of the Royal College of Anesthetists (FRCA) Examinations Regulations, published by Royal College of Anesthetists, it is recommended that candidates who fail the exam discuss with the tutor or trainer, and receive specific preparation or additional training before re-taking the exam. Similar suggestions are made for successful candidates, and they are recommended to review their feedback and work on their weaknesses within the scope of “ongoing professional development” with their tutors or trainers [16].

UEMS-CESMA also recommends sharing the results with the candidates and consulting the unsuccessful candidates after the exam [27]. Among the suggestions of Cascarini and Irani for the candidates preparing for the board exam is to examine and master the exam curriculum [60]. For example, they suggested that the candidate’s study not only form and function, but also cause and affect relationships in the preparation process and learn why they have failed. In our study, there were many question-expressions that met this standard, being mostly under the title of “Before exam/Preparation of the exam”.

Ideal evaluations are not always possible due to limitations in resources [13]. Naturally, for any activity to be feasible and of high utility, it is expected that the cost will not be high. This is also valid for evaluation, and for evaluation it is expected that the cost should be reasonable [12]. Considering the questions matched with cost in the checklist; examiners, question writers, staff, standardized patient (SP) trainings, all kinds of equipment and controls to be used for different purposes before and after the exam visits to the exam venue, meetings with different purposes before and after the exam, etc. are indispensable situations that require cost. We also found that steps that have costs are largely related to validity and reliability. With this in mind, the cost can be waived, since the aim is to perform an excellent exam. On the other side, some steps such as using computers instead of examiners can save costs.

Practicality is one of the most controversial topics in exam standards. Unfortunately, there is no language unity on the subject. Concepts such as practicality, practicability, usability, feasibility can be used together and intertwined for this purpose [20, 25, 59]. In our study, since the steps are handled in terms of easy to implement and apply, we preferred to use the word ‘practicality’. The vast majority of the steps desired to be carried out in our checklist are the practical steps. However, as in cost, some steps may not be practical even though they are mandatory. As in the question, "Is the examination carried out with a team of different people with different qualifications?” to find people with different qualifications is not practical although it has a positive role in the quality of the examination. This example also shows that the same step can make a contrast with each other in terms of standards, as it shows that the expectation of practicality can adversely affect the cost.

We think that the main limitation of our study may be the number of expert opinions received. Although it is hypothetically valid that more experts' opinions might have contributed more to the determination of the exam steps that lead us to perfection, it has been suggested that this constraint can be ignored by long-term experienced experts from different countries having international exam practices. Additionally, as this tool will be accessible on the web this will provide a platform to dynamically improve the checklist with the continuing contribution of different opinions and experiences. Our tool is focused on radiology. But we believe that it can be easily adapted to and be used by other specialties including not only the physicians but the other health professionals.

As a result, our tool has the properties of covering all the steps of a board exam, of being prepared and validated with the help of experienced specialist opinions, bringing explanations about each step, being prepared as yes no questions, steps being accessible on the web and associated with the exam and education standards. It should be accepted as an instructional tool that will be developed with continuous feedback that will be derived from practical applications. Quantitative data that will be obtained in these applications in line with our guide will increase the reproducibility of our tool.

We hope that it will be a useful guide for board exam organizers, exam staff and candidates who will take part in the exam.

## Data Availability

The datasets generated and/or analyzed during the current study are available in the link https://medinfo.deu.edu.tr/checklist/.

## Abbreviations

AAPM: American Association of Physicists in Medicine
ABIM: American Board of Internal Medicine
ABMS: American Board of Medical Specialties
ABR: American Board of Radiology
ACGME: Accreditation Council for Graduate Medical Education
ACR: American College of Radiology
AERA: American Educational Research Association
AMA: Radiology Department of American Medical Association
AMEE: Association for Medical Education in Europe
ARRS: American Roentgen Ray Association
ARS: American Radium Association
ASTRO: American Society for Radiation Oncology
AUR: Association of University Radiologists
CESMA: Council for European Medical Specialty Assessments
CME/CPD: Continued medical education/Continuing professional development
EDA: European Diploma of Anesthesiology
EDiR: European Diploma in Radiology
EPMA: European Postgraduate Medical Assessments
FRCA: Fellowship of the Royal College of Anesthetists
GMC: General Medical Council
MOC: Maintenance of certification programs
OLA: Online Longitudinal Assessment
OSCE: Objective structured clinical examination
RSNA: Radiological Society of North America
SIR: Society for Interventional Radiology
UEMS: European Union of Medical Specialists
USA: United States of America
WFME: World Federation for Medical Education
